# Type 2 Diabetes in Obese Individuals Is Associated With Central Adiposity and Atherogenic Dyslipidemia but Does Not Exacerbate Systemic Inflammation: A Cross-Sectional Study

**DOI:** 10.7759/cureus.97351

**Published:** 2025-11-20

**Authors:** Onur Gursoy, Enis Akca, Mustafa Oktay Tarhan, Metin Ozgen, Demet Yalcin Kehribar

**Affiliations:** 1 Internal Medicine, Tuzla Public Hospital, Istanbul, TUR; 2 Internal Medicine, Samsun Education and Research Hospital, Samsun, TUR; 3 Department of Internal Medicine, Dokuz Eylul University, Faculty of Medicine, Izmir, TUR; 4 Rheumatology, Izmir Ekonomi University Medical Point Hospital, Izmir, TUR

**Keywords:** atherogenic dyslipidemia, central adiposity, metabolic syndrome, neutrophil-to-lymphocyte ratio, obesity, systemic immune-inflammation index, systemic inflammation, type 2 diabetes mellitus, visceral fat, waist-to-hip ratio

## Abstract

Background: Obesity and type 2 diabetes mellitus (T2DM) are frequently coexisting conditions characterized by low-grade chronic inflammation. The primary objective of this study was to investigate the impact of T2DM, in the context of obesity, on body fat distribution, atherogenic lipid profiles, and systemic inflammatory markers derived from hematologic parameters.

Methodology: This cross-sectional study included a total of 171 participants with a body mass index (BMI) greater than 30 kg/m². Participants were divided into two groups: those with a diagnosis of T2DM (n = 86) and those without T2DM (n = 85). Between-group comparisons were performed for demographic characteristics, anthropometric measurements (waist-to-hip ratio (WHR), visceral adiposity score), lipid profiles (triglycerides, low-density lipoprotein (LDL)/triglyceride ratio), and systemic inflammatory markers derived from hematologic parameters, including the neutrophil-to-lymphocyte ratio (NLR), platelet-to-lymphocyte ratio (PLR), and systemic immune-inflammation index (SII). Appropriate parametric and nonparametric tests were used for statistical analyses.

Results: Although both groups exhibited comparable BMI values (p = 0.121), the diabetic cohort had a significantly higher WHR (0.97 ± 0.08 vs. 0.91 ± 0.10, p < 0.001). Metabolically, individuals with diabetes demonstrated elevated triglyceride levels (p = 0.023) and a lower LDL/triglyceride ratio (p = 0.003). Systemic inflammatory indices such as NLR, PLR, and SII were comparable between the two groups (all p > 0.05). Correlation analyses revealed that the relationship between visceral adiposity and inflammation was stronger in the non-diabetic group.

Conclusions: Our findings indicate that, in obese individuals, the presence of T2DM is associated not with a greater degree of overall adiposity, but rather with a distinct metabolic phenotype characterized by centralization of fat distribution and accentuated atherogenic dyslipidemia. The inability of commonly used systemic inflammatory markers to discriminate between groups may be attributed to a “ceiling effect” resulting from the elevated inflammatory background inherent to obesity and the potential “masking effect” of antidiabetic therapies with anti-inflammatory properties in the diabetic cohort. These results underscore the superiority of the WHR over BMI for cardiometabolic risk stratification in obese individuals and highlight the critical importance of treatment history when interpreting inflammatory indices.

## Introduction

Obesity and type 2 diabetes mellitus (T2DM) are now recognized as interrelated global epidemics. In 2021, an estimated 2.1 billion individuals worldwide were classified as overweight or obese, and this number is projected to rise to 3.8 billion by 2050 [1]. The increasing prevalence of obesity is not confined to developed nations but has also reached alarming proportions in developing countries, including Türkiye [1]. Beyond being a simple excess of body weight, obesity represents a major risk factor for numerous diseases such as T2DM, cardiovascular disorders, and several types of cancer [2].

Adipose tissue is not merely a passive energy reservoir but functions as an active endocrine organ that secretes a wide array of adipokines, cytokines, and chemokines [3]. In obesity, particularly with increased visceral fat, adipocytes secrete higher levels of pro-inflammatory cytokines such as interleukin-6 (IL-6) and tumor necrosis factor-alpha (TNF-α), while reducing the secretion of the anti-inflammatory adipokine adiponectin, thereby promoting inflammation [4]. Macrophage infiltration into adipose tissue and the phenotypic shift of macrophages from the anti-inflammatory M2 to the pro-inflammatory M1 subtype further amplify this inflammatory state. Visceral adipose tissue also contributes to hepatic inflammation by releasing free fatty acids and inflammatory mediators directly into the portal circulation [4]. These inflammatory processes are important in the development of dyslipidemia, insulin resistance, and thus in the pathogenesis of T2DM [3,4]. Moreover, T2DM itself represents an inflammatory condition characterized by immune dysregulation, hyperglycemia, and oxidative stress. Obesity and T2DM frequently coexist, and their concurrence may synergistically amplify cardiometabolic risk [5].

In assessing metabolic risk, it is increasingly recognized that the regional distribution of body fat is more critical than the total amount of adiposity. Unlike general adiposity indices such as body mass index (BMI), anthropometric measures including waist circumference and waist-to-hip ratio (WHR), as well as more specific parameters such as visceral adiposity score obtained via bioelectrical impedance analysis, provide a more accurate reflection of visceral adiposity and associated metabolic risk [6]. Furthermore, new, cost-effective inflammation markers that can be easily calculated from a complete blood count, such as neutrophil/lymphocyte ratio (NLR), platelet/lymphocyte ratio (PLR), and systemic immune-inflammation index (SII), are gaining importance in clinical practice due to their prognostic value [7].

Although the strong association between obesity, diabetes, and inflammation is well established, the extent to which the presence of diabetes modulates the inflammatory milieu and alters body fat distribution in obese individuals remains unclear. Accordingly, the aim of our study was to comparatively evaluate the impact of T2DM on visceral adiposity score, WHR, and a range of systemic inflammatory indices in obese individuals.

## Materials and methods

Study design and population

This cross-sectional study was conducted between December 2023 and January 2025 at the Obesity Outpatient Clinic of Dokuz Eylül University Faculty of Medicine. Individuals aged 18 years or older with a BMI greater than 30 kg/m², consistent with the definition of obesity, were included in the study. Subjects with a diagnosed active malignancy, known autoimmune or autoinflammatory disease, active infection at the time of evaluation, or current pregnancy were excluded. Based on these criteria, a total of 171 obese participants were enrolled, including 86 individuals with T2DM and 85 without T2DM.

The diagnosis of T2DM was confirmed according to medical records indicating an HbA1c value ≥6.5%, fasting blood glucose >126 mg/dL on different measurements, or active use of antidiabetic therapy.

The study protocol was approved by the institutional ethics committee of Dokuz Eylül University (approval number: 2024/01-12). All procedures were conducted in accordance with the principles of the Declaration of Helsinki.

Data collection and variables

All clinical and laboratory data of the participants were obtained retrospectively through the hospital’s electronic medical record system. Due to the retrospective nature of the study, detailed data regarding specific drug dosages and treatment durations (e.g., metformin, statins) could not be collected. Furthermore, data on disease duration and specific metrics of glycemic control (e.g., HbA1c) for the diabetic cohort were not available for this retrospective analysis.

Participant profile and clinical data

For each participant, demographic characteristics including age, sex, and comorbidity history were recorded in detail.

Anthropometric measurements and body composition

Body weight (kg) and height (m) were measured according to the standard protocols of the obesity outpatient clinic. BMI was calculated as weight divided by height squared (kg/m²).

Waist circumference was measured at the level of the umbilicus, and hip circumference was measured at the widest gluteal region with a tape measure applied horizontally, ensuring it was snug but did not compress the skin. Measurements were taken by trained clinic staff to minimize inter-rater variability. WHR was calculated from these measurements.

The visceral adiposity score was determined using a Tanita (Tanita Corporation, Tokyo, Japan) body composition analyzer based on the bioelectrical impedance analysis (BIA) method. All measurements were performed under standardized conditions - after a minimum of eight hours of fasting, with participants barefoot and wearing light clothing. The device integrates demographic parameters (age, sex, and height) with measured impedance values to calculate a visceral fat score ranging from 1 to 59, with values ≥13 indicating increased visceral adiposity.

Laboratory analyses

Laboratory parameters were obtained from routine blood test results recorded during the participants’ outpatient clinic visits. All blood samples were drawn following the eight-hour fast and processed according to the hospital's standardized laboratory protocols. The following parameters were documented: neutrophil, lymphocyte, monocyte, and platelet counts; mean platelet volume (MPV); ferritin; C-reactive protein (CRP); triglyceride; and low-density lipoprotein cholesterol (LDL-C) levels.

Using these primary data, the following indices were calculated: NLR, PLR, lymphocyte-to-monocyte ratio (LMR), and LDL/triglyceride ratio (LDL/TG).

The SII was calculated using the formula (platelet count × neutrophil count)/lymphocyte count and was used in statistical analyses.

Statistical analysis

Data processing and statistical analyses were performed using IBM SPSS Statistics version 24.0 (IBM Corp., Armonk, NY, USA). The normality assumption for all continuous variables was tested using the Shapiro-Wilk and Kolmogorov-Smirnov tests. Variables with a Gaussian distribution were reported as mean ± standard deviation, while non-normally distributed variables were expressed as median and interquartile range. Categorical data were presented as numbers and corresponding percentages. 

Differences in continuous variables between groups were determined by the independent samples t-test or the Mann-Whitney U test, depending on the data distribution. Specifically, the independent samples t-test was used for normally distributed continuous variables (e.g., age, anthropometric measures), while the Mann-Whitney U test was applied for non-normally distributed variables (e.g., inflammatory indices, lipid ratios). Relationships for categorical variables were examined using the Pearson Chi-square (χ²) test or Fisher's Exact test where appropriate. 

Based on the fulfillment of parametric assumptions, bivariate correlations were assessed with Pearson or Spearman correlation analyses. For all inferential tests, statistical significance was set at p<0.05. No adjustments were made for multiple comparisons.

## Results

A total of 171 obese participants with a BMI greater than 30 kg/m² were included in the study. Participants were divided into two groups: diabetic obese (n = 86; 63 females, 22 males) and non-diabetic obese (n = 85; 56 females, 30 males).

The mean age of the diabetic obese group (49.99 ± 10.39 years) was slightly higher than that of the non-diabetic group (46.86 ± 10.61 years), approaching statistical significance (p = 0.053). The groups were comparable in terms of sex distribution, BMI, waist circumference, and visceral fat score (all p > 0.05). However, the diabetic group exhibited a lower hip circumference (121.65 ± 10.56 cm) than the non-diabetic group (124.80 ± 10.04 cm; p = 0.047). The most prominent difference was observed in the WHR, a marker of central obesity, which was significantly higher in the diabetic group (0.97 ± 0.08) compared with the non-diabetic group (0.91 ± 0.10; p < 0.001).

Regarding comorbidities, the prevalence of hypertension (48 (55.8%) vs. 20 (23.5%), p < 0.001) and hyperlipidemia (42 (48.8%) vs. 24 (28.2%), p = 0.006) was significantly higher in the diabetic obese group. A summary of the demographic and anthropometric characteristics is presented in Table 1.

**Table 1:** Comparison of Demographic, Anthropometric, and Clinical Characteristics of the Participants Groups were compared using the Independent Samples t-Test and Chi-Square Test.

Variable	Diabetic Obese (n=86) (Mean ± SD)	Non-diabetic Obese (n=85) (Mean ± SD)	p-value
Demographic data			
Age (years)	49.99 ± 10.39	46.86 ± 10.61	0.053
Sex (Female/Male)	63 / 22	56 / 30	0.245
Anthropometric Measurements			
Body Mass Index (kg/m²)	38.16 ± 5.15	36.94 ± 5.08	0.121
Waist Circumference (cm)	118.43 ± 16.62	114.31 ± 14.82	0.089
Hip Circumference (cm)	121.65 ± 10.56	124.80 ± 10.04	0.047
Waist-to-Hip Ratio	0.97 ± 0.08	0.91 ± 0.10	<0.001
Visceral Adiposity Score	14.03 ± 5.05	12.76 ± 5.15	0.106
Comorbidities (n (%))			
Hypertension, n (%)	48 (%55.8)	20 (%23.5)	<0.001
Hyperlipidemia, n (%)	42 (%48.8)	24 (%28.2)	0.006
Chronic kidney disease, n (%)	10 (%11.6)	7 (%8.2)	0.458
Coronary artery disease, n (%)	6 (%7.0)	2 (%2.4)	0.152
Congestive Heart Failure, n (%)	3 (%3.5)	1 (%1.2)	0.317
Cerebrovascular Event, n (%)	2 (%2.3)	1 (%1.2)	0.567

No significant differences were observed between the two groups regarding key inflammatory markers, including NLR, PLR, ferritin, CRP, and SII (all p > 0.05). In the diabetic obese group, triglyceride levels were higher (186.97 ± 93.84 mg/dL) compared to the nondiabetic group (154.99 ± 88.31 mg/dL) (p=0.023). In parallel, the LDL/triglyceride ratio, an indicator of atherogenic dyslipidemia, was significantly lower in the diabetic group (0.83 ± 0.42) than in the non-diabetic group (1.04 ± 0.51; p = 0.003). The comparison of laboratory and inflammatory parameters between the groups is summarized in Table 2.

**Table 2:** Comparison of Laboratory Values and Inflammatory Markers Between Groups Statistically significant p-values (p<0.05) are shown in bold. MPV: mean platelet volume, LDL: low-density lipoprotein, CRP: C-reactive protein.

Variable	Unit	Diabetic Obese (n=86) (Mean ± SD)	Non-diabetic Obese (n=85) (Mean ± SD)	p-value
Hemogram				
Neutrophil	10³/μL	4.73 ± 1.43	4.49 ± 1.39	0.265
Lymphocyte	10³/μL	2.50 ± 0.74	2.40 ± 0.68	0.345
Platelet	10³/μL	286.36 ± 77.96	280.07 ± 61.91	0.560
Monocyte	10³/μL	0.55 ± 0.17	0.57 ± 0.15	0.386
MPV	fL	8.91 ± 0.94	8.75 ± 0.86	0.264
Lipid Profile				
LDL Cholesterol	mg/dL	126.47 ± 33.76	133.62 ± 36.57	0.186
Triglyceride	mg/dL	186.97 ± 93.84	154.99 ± 88.31	0.023
Inflammatory Markers				
Neutrophil/Lymphocyte Ratio	-	2.02 ± 0.80	1.97 ± 0.72	0.679
Platelet/Lymphocyte Ratio	-	118.76 ± 39.65	124.70 ± 43.65	0.353
Lymphocyte/Monocyte Ratio	-	4.82 ± 1.79	4.37 ± 1.37	0.071
LDL/Triglyceride Ratio	-	0.83 ±0.42	1.04 ±0.51	0.003
Systemic Immune-Inflammation Index	-	560.60 ± 219.29	555.31 ± 256.88	0.885
Ferritin	ng/mL	50.40 ± 66.22	45.51 ± 49.78	0.586
CRP	mg/dL	7.10 ± 6.11	7.82 ± 11.53	0.612

Correlation analysis performed in the overall study population (n = 171) confirmed the expected strong relationships between visceral adiposity indices and anthropometric measures (Table 3). The visceral fat score showed strong positive correlations with waist circumference (r = 0.782, p < 0.001) and BMI (r = 0.670, p < 0.001), while the WHR also demonstrated a strong correlation with waist circumference (r = 0.787, p < 0.001). Both the visceral fat score (r = 0.414, p < 0.001) and WHR (r = 0.389, p < 0.001) exhibited significant positive correlations with ferritin, an acute-phase reactant. Additionally, higher indices of obesity and visceral adiposity were consistently negatively correlated with the LDL/triglyceride ratio, a surrogate marker of atherogenic dyslipidemia (r = −0.230 for BMI; r = −0.222 for WHR).

**Table 3:** Significant Correlations of Visceral Adiposity Markers with Anthropometric and Inflammatory Parameters in the Entire Group (n=171) Spearman rank correlation coefficient (r) is shown. Only statistically significant correlations are presented. LDL: low-density lipoprotein, CRP: C-reactive protein.

	Visceral Fat Score	Waist-to-Hip Ratio	BMI
Variable	Correlation (r)	Correlation (r)	Correlation (r)
Body Mass Index	0.670 (p<0.001)	0.396 (p<0.001)	-
Waist Circumference	0.782 (p<0.001)	0.787 (p<0.001)	0.750 (p<0.001)
Hip Circumference	0.444 (p<0.001)	-	0.736 (p<0.001)
Ferritin	0.414 (p<0.001)	0.389 (p<0.001)	-
Monocyte	0.199 (p=0.009)	0.247 (p=0.001)	-
Neutrophil	-	0.176 (p=0.021)	0.248 (p=0.001)
CRP	-	-	0.196 (p=0.010)
Triglyceride	-	0.166 (p=0.030)	-
LDL/Triglyceride Ratio	-0.160 (p=0.038)	-0.222 (p=0.003)	-0.230 (p=0.002)

To further investigate how the presence of diabetes modulates these associations, subgroup analyses were conducted (Table 4). Notable differences were observed between groups. The relationship between inflammation and visceral adiposity was more pronounced in the non-diabetic obese group, where ferritin correlated strongly with both the visceral fat score (r = 0.588, p < 0.001) and WHR (r = 0.498, p < 0.001), whereas in the diabetic group, these correlations were weaker (r = 0.289 and r = 0.324, respectively).

**Table 4:** Key Correlations of Ferritin and Visceral Fat Score in Subgroups Spearman rank correlation coefficient (r) is shown. Only selected, statistically significant correlations are presented to highlight differences between the groups. LDL: low-density lipoprotein, CRP: C-reactive protein.

	Diabetic Obese Group (n=86)	Non-diabetic Obese Group (n=85)
Variables	Correlation (r)	Correlation (r)
Ferritin vs:		
Waist-to-Hip Ratio	0.324 (p=0.002)	0.498 (p<0.001)
Visceral Fat Score	0.289 (p=0.007)	0.588 (p<0.001)
Triglyceride	0.345 (p=0.001)	Not Significant
Visceral Fat Score vs:		
Waist-to-Hip Ratio	0.842 (p<0.001)	0.530 (p<0.001)
BMI vs:		
CRP	0.392 (p<0.001)	Not Significant
LDL/Triglyceride Ratio	Not Significant	-0.286 (p=0.008)

Distinct metabolic and inflammatory patterns were identified within each group. The positive correlation between BMI and CRP was significant only in the diabetic group (r = 0.392, p < 0.001), whereas the negative correlation between BMI and the LDL/triglyceride ratio reached significance only in the non-diabetic group (r = −0.286, p = 0.008). Moreover, a strong positive correlation was found between the visceral fat score and WHR in the diabetic group (r = 0.842, p < 0.001).

## Discussion

This study comparatively examined the associations of T2DM with body fat distribution, dyslipidemia profile, metabolic parameters, and systemic inflammatory markers in the context of obesity. The key findings of our investigation reveal a distinct dichotomy between diabetic and non-diabetic obese cohorts. On one hand, the diabetic state was clearly associated with central obesity (reflected by a higher WHR) and atherogenic dyslipidemia (elevated triglycerides and reduced LDL/triglyceride ratio). On the other hand, despite the well-established proinflammatory nature of both conditions, the two groups were comparable in terms of widely used systemic inflammatory indices such as NLR, PLR, SII, and CRP.

The most prominent finding of our study is the significantly higher WHR in the diabetic obese group compared to the nondiabetic obese group. Interestingly, this difference emerged despite similar BMI and waist circumference values between groups, primarily due to the significantly lower hip circumference observed in the diabetic cohort. These results suggest that the diabetic state is associated with a phenotype characterized by preferential fat accumulation in the abdominal region rather than in peripheral (gluteofemoral) depots, which is known to be one of the strongest determinants of cardiometabolic risk [8].

This observation strongly supports the extensive body of literature advocating the superiority of central adiposity metrics over general obesity indices for predicting metabolic risk [6,9]. Although BMI remains a practical and widely used measure, it fails to distinguish between fat compartments. Our data indicate that, at comparable levels of general obesity, the distribution of adipose tissue, rather than its total amount, is what most clearly differentiates diabetic from non-diabetic individuals. A high WHR serves as a clinical surrogate of increased visceral adipose tissue, which represents a metabolically active and highly pathogenic fat depot [10].

Unlike subcutaneous fat, visceral adipose tissue (VAT) exerts profound metabolic effects through the direct release of free fatty acids (FFAs) into the portal circulation, thereby promoting hepatic and peripheral insulin resistance - the hallmark of T2DM. VAT also contributes to metabolic dysfunction by secreting pro-inflammatory adipokines such as IL-6 and TNF-α, while simultaneously reducing the secretion of protective adipokines such as adiponectin [10,11]. In the presence of insulin resistance, the increased flow of free fatty acids to the liver triggers hepatic very low-density lipoprotein (VLDL) production, while reduced lipoprotein lipase activity slows the clearance of triglyceride-rich lipoproteins, leading to hypertriglyceridemia [12].

The significantly higher triglyceride levels and the correspondingly lower LDL/triglyceride ratio observed in the diabetic obese group indicate the presence of atherogenic dyslipidemia, a high-risk lipid triad commonly associated with T2DM [13]. A low LDL/triglyceride ratio reflects the predominance of small, dense LDL (sdLDL) particles, which are more susceptible to oxidation, penetrate the arterial wall more readily, and have markedly greater atherogenic potential [14]. These findings provide a clear mechanistic explanation for the significantly higher prevalence of hypertension and hyperlipidemia in the diabetic obese population, elucidating a central pathway underlying their increased cardiovascular risk.

Central adiposity, as reflected by WHR, not only drives insulin resistance but also shows a stronger association with β-cell dysfunction compared with other obesity metrics [15]. The transition from insulin resistance to overt diabetes occurs when β-cells fail to maintain adequate compensatory insulin secretion. Therefore, the higher WHR observed in the diabetic group likely reflects a phenotype characterized by both impaired insulin sensitivity and defective insulin secretion, underscoring WHR’s strong predictive value for T2DM development [15].

Moreover, the lower hip circumference in the diabetic group may indicate a relative depletion of gluteofemoral fat, which has been shown to exert metabolically protective effects [16]. This suggests that diabetes is not only associated with the accumulation of visceral fat, but also with the inability to appropriately store lipids in “safe” subcutaneous depots. The “lipid overflow” hypothesis posits that this impaired storage capacity leads to ectopic fat deposition in visceral organs, further exacerbating insulin resistance [17].

These mechanisms collectively reinforce the clinical value of WHR as a simple yet powerful composite marker, capable of capturing multiple interrelated risk factors - visceral adiposity, subcutaneous abdominal fat, and depletion of protective gluteofemoral depots - within a single measurement. Notably, while the visceral fat score did not differ between groups in our study, the WHR remained significantly higher in the diabetic cohort. This discrepancy suggests that WHR may provide a more integrative assessment of body fat distribution and metabolic risk, maintaining its clinical utility even in the era of advanced body composition technologies such as BIA.

One of the most unexpected findings of our study was the similarity in systemic inflammatory markers, including NLR, PLR, SII, and CRP, between diabetic and non-diabetic obese individuals. Although no previous study, to our knowledge, has simultaneously compared all of these indices in such cohorts, earlier research similarly reported comparable CRP levels between diabetic and non-diabetic obese patients [18]. Elevated NLR and SII levels have been associated with an increased risk and prevalence of T2DM [19,20]. However, our results suggest that in an already obese population, these commonly used and cost-effective inflammatory indices may be insufficient to distinguish between individuals with and without T2DM.

Several possible explanations may account for this observation. First, severe obesity itself may have created a “ceiling effect” for these markers. Obesity is well known to elevate leukocyte and neutrophil counts as well as derived indices such as NLR and SII [21,22]. Given that all participants in our study had a BMI greater than 30 kg/m², the chronic, obesity-related inflammation may have already activated these pathways to their maximal extent, masking any additional inflammatory contribution from diabetes. Consequently, our non-diabetic obese group cannot be regarded as a “healthy control” population, but rather as individuals situated along the continuum of metabolic disease characterized by obesity-driven inflammation.

Second, hematologic ratios such as NLR and SII are non-specific markers reflecting the balance of circulating immune cells. While useful for systemic inflammatory assessment, they may lack the sensitivity to detect localized or tissue-level inflammation occurring within metabolically active organs such as visceral adipose tissue, liver, and pancreatic islets - sites central to the pathogenesis of T2DM.

Third, the potential confounding effect of pharmacological therapy in the diabetic cohort must be considered. In our study, the diabetic obese group exhibited significantly higher rates of hypertension (48 (55.8%) vs. 20 (23.5%) and hyperlipidemia (42 (48.8%) vs. 24 (28.2%), suggesting that a substantial proportion were receiving antihypertensive and lipid-lowering medications, particularly statins. Moreover, it is reasonable to assume that metformin, the first-line therapy for T2DM, was widely used among these patients. Both classes of drugs are well known for their anti-inflammatory properties.

Metformin has been shown to inhibit the NF-κB signaling pathway, thereby reducing systemic inflammatory mediators such as CRP, TNF-α, and IL-6 beyond its glucose-lowering effects [23,24]. Likewise, statins, independent of their lipid-lowering action, significantly decrease CRP and circulating cytokine levels [25]. Within this context, the absence of detectable differences in inflammatory markers between groups may not necessarily reflect biological equivalence, but rather a potential therapeutic masking effect. It is plausible that the anti-inflammatory treatments likely received by the diabetic participants may have suppressed their baseline - potentially higher-inflammatory burden to a level statistically indistinguishable from that observed in the non-diabetic obese group. However, this interpretation remains speculative, as we lacked data on specific medications, dosages, or treatment durations. An alternative explanation, alongside the 'ceiling effect,' is that these non-specific, systemic markers (NLR, CRP) are simply not sensitive enough to detect the specific, low-grade inflammation associated with T2DM pathogenesis once high-grade obesity-driven inflammation is already present.

Taken together, these results suggest that commonly used systemic inflammatory markers such as NLR and CRP may have limited discriminative power in differentiating inflammatory status between treated diabetic and non-diabetic obese individuals.

Our correlation analysis revealed a nuanced pattern suggesting that the nature of inflammation may shift in the presence of diabetes. The strong correlation observed between visceral adiposity and serum ferritin in the non-diabetic group was markedly attenuated in the diabetic group. This attenuation may indirectly reflect the suppressive effect of antidiabetic therapy on obesity-related chronic inflammation. Serum ferritin, beyond its role as an indicator of iron stores, functions as an acute-phase reactant elevated during inflammation and oxidative stress and is closely linked to visceral adiposity [26]. In non-diabetic obesity, the robust association between visceral fat and ferritin likely reflects a state of “metaflammation”, in which dysfunctional adipose tissue serves as the primary driver of systemic inflammation [27].

In the prediabetic state, dysfunctional visceral adipose tissue constitutes the predominant source of inflammatory signaling. However, once T2DM develops, new pro-inflammatory stimuli, such as chronic hyperglycemia, advanced glycation end-products (AGEs), and systemic oxidative stress, emerge as major contributors [28]. In our study, a significant positive correlation between BMI and CRP was observed only in the diabetic group. This finding suggests that in this more generalized inflammatory milieu, the total metabolic load represented by BMI becomes more strongly associated with global markers such as CRP [29]. Collectively, these findings imply that as T2DM becomes established, the inflammatory landscape evolves, from one primarily driven by dysfunctional visceral fat to a more complex and systemic process influenced by overall metabolic burden [29].

The clinical implications of these findings are substantial. They emphasize that clinicians evaluating obese patients should not rely solely on BMI for diabetes risk assessment. Simple and cost-effective anthropometric measures such as WHR may provide greater predictive value for visceral adiposity and associated cardiometabolic risk. Furthermore, normal NLR or CRP levels in a diabetic obese patient, particularly one receiving metformin and/or statin therapy, should not be interpreted as evidence of an absence of inflammation. This underscores the importance of considering the patient’s pharmacological regimen when interpreting inflammatory markers in clinical practice.

The current findings also open new directions for future research. Prospective studies involving medication-naïve or newly diagnosed individuals, or those with standardized treatment protocols, are needed to isolate the true biological interaction between obesity and T2DM on inflammation, free from confounding therapeutic effects. In addition, quantifying specific cytokines and adipokines, such as adiponectin, IL-6, and TNF-α, would yield deeper mechanistic insights into the inflammatory and anti-inflammatory balance that distinguishes these metabolic states.

Several limitations must be acknowledged when interpreting our results. First, due to the cross-sectional design, causality cannot be inferred. Second, as a single-center study, generalizability may be limited. Third, our study population was drawn from a specialized obesity clinic, which may introduce selection bias and limit generalizability to the broader obese population. Fourth, one of the most significant limitations is the lack of data on diabetes duration, glycemic control (i.e., HbA1c levels), and the potential confounding effect of anti-inflammatory medications such as metformin and statins, which were presumed to be widely used among diabetic participants. The lack of detailed data on dosage and treatment duration precluded a precise assessment of their influence on inflammatory indices. Fifth, we did not apply corrections for multiple comparisons, which increases the risk of Type I errors, although our most significant findings (e.g., WHR) had highly significant p-values. Sixth, BIA measurements are known to have limitations in accuracy in populations with severe obesity, although we attempted to standardize the procedure. Finally, the absence of statistically significant differences in inflammatory markers may also reflect insufficient statistical power (Type II error) to detect small between-group effects.

## Conclusions

In conclusion, our study demonstrates that among obese individuals, the presence of T2DM is associated not with greater overall adiposity, but with a distinct metabolic phenotype characterized by increased central obesity and potentially atherogenic dyslipidemia. The inability of commonly used systemic inflammatory markers to distinguish between diabetic and non-diabetic obesity likely reflects both the high inflammatory baseline inherent to obesity and the masking effects of anti-inflammatory therapies in the diabetic cohort. This finding must be interpreted with caution.

These findings underscore the clinical importance of shifting the focus beyond BMI when assessing diabetes risk in obese patients, emphasizing the utility of WHR as a marker of central adiposity and metabolic risk. Furthermore, the results highlight that interpretation of inflammatory status should always consider the patient’s current pharmacological treatment, which may significantly modulate systemic inflammatory markers.

## References

[1] (2025). Global, regional, and national prevalence of adult overweight and obesity, 1990-2021, with forecasts to 2050: a forecasting study for the Global Burden of Disease Study 2021. Lancet.

[2] Bae JP, Nelson DR, Boye KS, Mather KJ (2025). Prevalence of complications and comorbidities associated with obesity: a health insurance claims analysis. BMC Public Health.

[3] Wang S, Liu Y, Chen J, He Y, Ma W, Liu X, Sun X (2023). Effects of multi-organ crosstalk on the physiology and pathology of adipose tissue. Front Endocrinol (Lausanne).

[4] Turner L, Wanasinghe AI, Brunori P, Santosa S (2025). Is adipose tissue inflammation the culprit of obesity-associated comorbidities?. Obes Rev.

[5] Caturano A, Rocco M, Tagliaferri G (2025). Oxidative stress and cardiovascular complications in type 2 diabetes: from pathophysiology to lifestyle modifications. Antioxidants (Basel).

[6] Tuglo LS (2022). Comparison of adiposity anthropometric indices and their associations with visceral fat levels determined by bioelectrical impedance analysis among diabetic patients. Sci Rep.

[7] Ke J, Qiu F, Fan W, Wei S (2023). Associations of complete blood cell count-derived inflammatory biomarkers with asthma and mortality in adults: a population-based study. Front Immunol.

[8] Piché ME, Tchernof A, Després JP (2020). Obesity phenotypes, diabetes, and cardiovascular diseases. Circ Res.

[9] Khan I, Chong M, Le A (2023). Surrogate adiposity markers and mortality. JAMA Netw Open.

[10] Neeland IJ, Ross R, Després JP (2019). Visceral and ectopic fat, atherosclerosis, and cardiometabolic disease: a position statement. Lancet Diabetes Endocrinol.

[11] Tchernof A, Després JP (2013). Pathophysiology of human visceral obesity: an update. Physiol Rev.

[12] Taskinen MR, Borén J (2015). New insights into the pathophysiology of dyslipidemia in type 2 diabetes. Atherosclerosis.

[13] Manoria PC, Chopra HK, Parashar SK, Dutta AL, Pinto B, Mullasari A, Prajapati S (2013). The nuances of atherogenic dyslipidemia in diabetes: focus on triglycerides and current management strategies. Indian Heart J.

[14] Ouchi G, Komiya I, Taira S, Wakugami T, Ohya Y (2022). Triglyceride/low-density-lipoprotein cholesterol ratio is the most valuable predictor for increased small, dense LDL in type 2 diabetes patients. Lipids Health Dis.

[15] Liu N, Wang B, Zhang G (2025). Waist-to-hip ratio better reflect beta-cell function and predicts diabetes risk in adult with overweight or obesity. Ann Med.

[16] Manolopoulos KN, Karpe F, Frayn KN (2010). Gluteofemoral body fat as a determinant of metabolic health. Int J Obes (Lond).

[17] Virtue S, Vidal-Puig A (2010). Adipose tissue expandability, lipotoxicity and the metabolic syndrome--an allostatic perspective. Biochim Biophys Acta.

[18] Fronczyk A, Molęda P, Safranow K, Piechota W, Majkowska L (2014). Increased concentration of C-reactive protein in obese patients with type 2 diabetes is associated with obesity and presence of diabetes but not with macrovascular and microvascular complications or glycemic control. Inflammation.

[19] Guo X, Zhang S, Zhang Q (2015). Neutrophil:lymphocyte ratio is positively related to type 2 diabetes in a large-scale adult population: a Tianjin Chronic Low-Grade Systemic Inflammation and Health cohort study. Eur J Endocrinol.

[20] Nie Y, Zhou H, Wang J, Kan H (2023). Association between systemic immune-inflammation index and diabetes: a population-based study from the NHANES. Front Endocrinol (Lausanne).

[21] Zhou Y, Wang Y, Wu T, Zhang A, Li Y (2024). Association between obesity and systemic immune inflammation index, systemic inflammation response index among US adults: a population-based analysis. Lipids Health Dis.

[22] Furuncuoğlu Y, Tulgar S, Dogan AN, Cakar S, Tulgar YK, Cakiroglu B (2016). How obesity affects the neutrophil/lymphocyte and platelet/lymphocyte ratio, systemic immune-inflammatory index and platelet indices: a retrospective study. Eur Rev Med Pharmacol Sci.

[23] Karbalaee-Hasani A, Khadive T, Eskandari M (2021). Effect of metformin on circulating levels of inflammatory markers in patients with type 2 diabetes: a systematic review and meta-analysis of randomized controlled trials. Ann Pharmacother.

[24] Moiseeva O, Deschênes-Simard X, St-Germain E (2013). Metformin inhibits the senescence-associated secretory phenotype by interfering with IKK/NF-κB activation. Aging Cell.

[25] Ridker PM, Danielson E, Fonseca FA (2008). Rosuvastatin to prevent vascular events in men and women with elevated C-reactive protein. N Engl J Med.

[26] Iwasaki T, Nakajima A, Yoneda M (2005). Serum ferritin is associated with visceral fat area and subcutaneous fat area. Diabetes Care.

[27] Ding X, Bian N, Wang J, Chang X, An Y, Wang G, Liu J (2023). Serum ferritin levels are associated with adipose tissue dysfunction-related indices in obese adults. Biol Trace Elem Res.

[28] Kuryłowicz A, Koźniewski K (2020). Anti-inflammatory strategies targeting metaflammation in type 2 diabetes. Molecules.

[29] Sam S, Haffner S, Davidson MH (2009). Relation of abdominal fat depots to systemic markers of inflammation in type 2 diabetes. Diabetes Care.

